# The Quantum Paradox in Pharmaceutical Science: Understanding Without Comprehending—A Centennial Reflection

**DOI:** 10.3390/ijms26104658

**Published:** 2025-05-13

**Authors:** Sarfaraz K. Niazi

**Affiliations:** College of Pharmacy, University of Illinois, Chicago, IL 60012, USA; sniazi3@uic.edu; Tel.: +1-312-297-0000

**Keywords:** quantum tunneling, Schrödinger equation, Heisenberg uncertainty principle, Boltzmann distribution, enzyme catalysis, drug design, proton transfer, computational chemistry, quantum biochemistry

## Abstract

The Schrödinger equation, Heisenberg’s uncertainty principles, and the Boltzmann constant represent transformative scientific achievements, the impacts of which extend far beyond their original domain of physics. As we celebrate the centenary of these fundamental quantum mechanical formulations, this review examines their evolution from abstract mathematical concepts to essential tools in contemporary drug discovery and development. While these principles describe the behavior of subatomic particles and molecules at the quantum level, they have profound implications for understanding biological processes such as enzyme catalysis, receptor–ligand interactions, and drug–target binding. Quantum tunneling, a direct consequence of these principles, explains how some reactions occur despite classical energy barriers, enabling novel therapeutic approaches for previously untreatable diseases. This understanding of quantum mechanics from 100 years ago is now creating innovative approaches to drug discovery with diverse prospects, as explored in this review. However, the fact that the quantum phenomenon can be described but never understood places us in a conundrum with both philosophical and ethical implications; a prospective and inconclusive discussion of these aspects is added to ensure the incompleteness of the paradigm remains unshifted.

## 1. Introduction: The Quantum Revolution in Biology and Medicine

When Erwin Schrödinger published “Quantisierung als Eigenwertproblem” (Quantization as an Eigenvalue Problem) in January 1926 [1], and Werner Heisenberg formulated his uncertainty principle in 1927 [2], few could have envisioned the profound impact these theoretical physics concepts would eventually have on drug discovery and medicine. These works emerged from a period of extraordinary scientific ferment when quantum mechanics transformed our understanding of the physical world at its most fundamental level. What makes this centenary particularly significant is the historical milestone and how these theoretical frameworks have evolved into practical tools that drive innovation in pharmaceutical science. As Schrödinger later explored in his influential 1944 book “What is Life?” [3], quantum mechanical principles might be essential to understanding biological processes. This prescient insight established a conceptual bridge between quantum physics and biology that continues to yield discoveries today.

Though developed independently, Heisenberg’s matrix mechanics and Schrödinger’s wave equation represent complementary aspects of quantum theory [4]. The Schrödinger equation provides a mathematical description of how quantum states evolve, while Heisenberg’s uncertainty principle establishes fundamental limits on simultaneously knowing complementary properties of quantum systems. Together, they form the foundation for modern drug design and development computational approaches. Computational limitations significantly delayed the practical application of quantum mechanical principles in drug discovery. It was not until the 1950s that the first pioneering work in quantum biochemistry was conducted by Bernard and Alberte Pullman [5,6], who founded this field through their applications of quantum chemistry to biological problems. Their groundbreaking work on predicting the carcinogenic properties of aromatic hydrocarbons represented one of the first successful applications of quantum principles to medicine [7].

The widespread practical application of quantum mechanics in drug discovery had to wait until the 1990s, when computational power finally became sufficient for more complex calculations and software platforms like Gaussian became accessible to pharmaceutical researchers [8]. Today, quantum mechanical approaches are indispensable tools in rational drug design, enabling researchers to understand drug–target interactions at unprecedented levels of detail and to predict the properties of new therapeutic candidates with increasing accuracy. This review examines the historical development, current applications, and prospects of quantum mechanical principles in drug discovery, highlighting how concepts formulated a century ago continue to drive innovation in pharmaceutical science.

## 2. Theoretical Foundations: From Physics to Pharmaceutical Science

### 2.1. The Schrödinger Equation and Its Significance

The Schrödinger equation represents one of the most fundamental equations in quantum mechanics. In its time-dependent form, it describes how the quantum state of a physical system changes over time:

Time-Dependent Schrödinger Equation:

iħ ∂Ψ(r, t)/∂t = Ĥ Ψ(r, t)

where i: imaginary unit; ħ: reduced Planck constant; Ψ(r, t): wavefunction; depends on position r and time t); Ĥ: Hamiltonian operator (represents total energy).

Time-Independent Schrödinger Equation:

Ĥ ψ(r) = E ψ(r)

where ψ(r): stationary state wavefunction (depends only on position r); E: energy eigenvalue corresponding to ψ(r); Ĥ: Hamiltonian operator (includes kinetic and potential energy).

The wave function contains all possible information about a quantum system, with its square representing probability distributions. This probabilistic interpretation represents a fundamental departure from classical deterministic physics and has profound implications for understanding molecular interactions at the atomic level [9]. The Schrödinger equation enables the calculation of electron distributions, molecular orbitals, and energy states—all critical factors in drug–target interactions. By solving this equation (or approximations of it) for molecular systems, computational chemists can predict how drugs might bind to their targets, the stability of these interactions, and the energy changes involved in various molecular transformations [10].

### 2.2. Heisenberg’s Uncertainty Principle and Its Implications

Heisenberg’s uncertainty principle establishes fundamental limits on the precision with which complementary variables, such as position and momentum, can be known simultaneously:

ΔxΔp≥2ℏ

where Δx is the uncertainty in position; Δp is the uncertainty in momentum; ℏ is the reduced Planck constant

In the context of drug discovery, this principle has several important implications. First, it establishes theoretical limits on the precision of molecular modeling, particularly regarding the exact positions and momenta of atoms in a molecular system. This inherent uncertainty must be accounted for in computational approaches to drug design, often through statistical and probabilistic methods [11]. The uncertainty principle also influences our understanding of transition states in chemical reactions relevant to drug action and metabolism. It explains why perfect predictions of molecular behavior are impossible and why experimental validation remains essential even with the most sophisticated computational models [12].

### 2.3. The Boltzmann Distribution and Quantum Thermodynamics

A crucial concept often overlooked in discussions of quantum applications to biology is the Boltzmann distribution, which gives the probability of a particular state as a function of energy and temperature:

P(Ei)=∑je−Ej/kTe−Ei/kT

where P(Ei) is the probability of a state with energy Ei; k is the Boltzmann constant; T is the temperature

**Figure 1:**
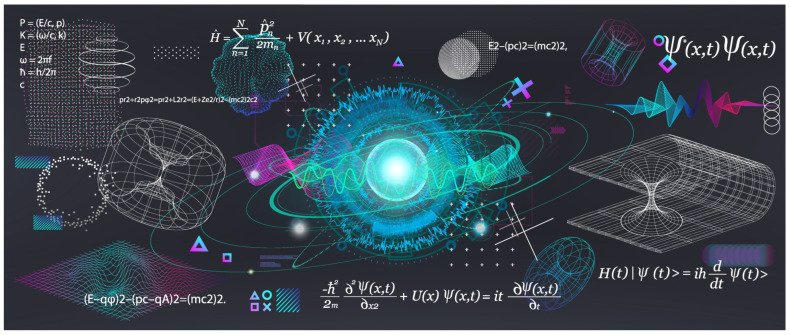
A stylized visualization of quantum mechanics concepts featuring various mathematical equations, geometric representations, and wave-like patterns. On the left side, several fundamental physics relationships are listed: P = (E/c, *p*)—relating momentum to energy and classical momentum; K = (ω/c, *k*)—wave vector relation; E—energy; ω = 2πf—angular frequency; ħ = h/2π—reduced Planck constant; c—speed of light. In the center-top, the Hamiltonian operator equation: Ĥ = ∑ (*n* = 1 to N) [p̂²ₙ/2m_n_] + V(x_1_, x_2_, …, x_n_) This represents the total energy operator of a quantum system with kinetic and potential energy terms. The time-dependent Schrödinger equation appears in the bottom right: [−ħ^2^/2 m·∂^2^ψ(x, t)/∂x^2^ + U(x)ψ(x, t)] = iħ·∂ψ(x, t)/∂t. This is the fundamental equation describing how quantum states evolve. The right side shows the Dirac notation of the Schrödinger equation: H(t)|ψ(t)⟩ = iħ·d/dt·ψ(t)⟩. The wavefunction notation ψ(x, t)ψ(x, t) appears in the upper right, representing probability amplitudes. These visualizations of quantum concepts such as wavefunction representations shown as oscillating waves; the orbital-like structures and energy level representations where a bright central blue-white sphere possibly representing a quantum particle or probability density; the wireframe geometric structures showing mathematical spaces (torus, wormhole-like structure). Additional equations E^2^ − (pc)^2^ = (mc^2^)^2^—the relativistic energy-momentum relation and p^2^ + 2pω^2^ = p^2^ + 2r^2^ = (E + Zℯ^2^/r)^2^ − (mc^2^)^2^c^2^—representing a quantum system with interactions. The overall composition blends mathematical formalism with artistic visualization of quantum mechanical concepts, including wave-particle duality, probability distributions, and quantum states.

This distribution is essential for understanding how quantum effects manifest in thermal biological environments. At physiological temperatures, many quantum effects might be expected to be washed out by thermal fluctuations. However, evidence increasingly suggests that specific biological systems have evolved to protect and harness quantum phenomena even in warm, wet environments [13]. The interplay between quantum mechanics and thermal statistics creates a rich landscape for understanding molecular interactions in biological systems, including how drugs interact with their targets under physiological conditions [14]. Figure 1 summarizes the interaction among several quantum mechanics paradoxes.

## 3. Bridging Quantum and Molecular Scales

A critical conceptual challenge in applying quantum mechanics to drug discovery is understanding how quantum effects, which primarily occur at atomic and subatomic scales, influence the behavior of larger molecular systems relevant to biology and medicine. Quantum mechanical effects such as tunneling, superposition, and entanglement are fundamental phenomena of elementary particles, atoms, and their constituents—not of large molecules as complete entities [15]. However, these quantum effects at the elemental level propagate upward to influence molecular behavior in several key ways.

### 3.1. Electron Distributions and Chemical Bonding

Molecular structure and reactivity are ultimately determined by electron distributions, which are inherently quantum mechanical. The Schrödinger equation describes how electrons behave in atoms and molecules, forming the quantum foundation for chemical bonding [16]. Consider the hydrogen bond, which is crucial in protein folding and drug–target interactions. While the hydrogen bond is often modeled using classical electrostatics, its strength, and directionality can only be accurately predicted by accounting for the quantum mechanical distribution of electrons around the hydrogen atom. In the case of the antibiotic vancomycin, its binding to bacterial cell wall components depends critically on five hydrogen bonds whose strength emerges from quantum effects in electron density distribution [17]. Classical mechanics alone cannot explain the observed binding energies without incorporating these quantum-derived electron distributions.

Similarly, the π-stacking interactions that stabilize many drug-aromatic amino acid interactions (as seen in histone deacetylase inhibitors) depend on quantum mechanical electron delocalization that cannot be derived from classical physics [18]. The electron densities that determine these interactions must be calculated using the Schrödinger equation, even though the overall molecular motion follows classical trajectories.

### 3.2. Localized Quantum Events in Large Systems

In biological macromolecules such as enzymes, quantum effects often occur locally in specific regions while the rest of the system behaves classically. Soybean lipoxygenase catalyzes hydrogen transfer with a kinetic isotope effect (KIE) of approximately 80, far exceeding the maximum value of ~7 predicted by classical transition state theory [19]. This enormous KIE indicates that hydrogen tunnels through, rather than over, the energy barrier. Importantly, this tunneling is a quantum event localized to the transferred hydrogen atom—the remainder of the enzyme (thousands of atoms) behaves classically. The drug design implications are significant: lipoxygenase inhibitors engineered to disrupt optimal tunneling geometries can achieve greater potency than those designed solely on classical considerations [20].

Another striking example occurs in DNA, where proton tunneling affects tautomerization rates between canonical and rare tautomeric forms of nucleobases. While rare (occurring approximately once per 10,000 to 100,000 base pairs), these quantum events can cause spontaneous mutations [21]. Some DNA repair enzyme inhibitors developed as anticancer agents target processes that correct these quantum-induced mutations [22].

### 3.3. Quantum-Classical Interface in Computational Drug Design

The transition from quantum to classical behavior in biological systems is not abrupt but gradual and context-dependent, creating a computational hierarchy in drug design. In the structure-based design of HIV protease inhibitors, a multi-scale approach demonstrates this quantum-classical interface [23]. The critical interactions between the catalytic aspartate residues and the inhibitor (such as darunavir) involve proton transfer energetics that require quantum mechanical calculations. However, modeling the entire protein-inhibitor complex quantum mechanically would be computationally prohibitive. Instead, QM/MM methods are employed where the active site and inhibitor (~50–100 atoms) are treated using quantum mechanics, while the rest of the protein and solvent (~10,000+ atoms) are treated with classical mechanics. Information flows between these regions, with quantum effects influencing classical behavior and vice versa [24].

This hierarchical approach has developed second-generation HIV protease inhibitors with picomolar binding affinities and reduced susceptibility to resistance mutations [25]. The quantum calculations reveal subtle electronic effects that classical force fields miss, while the classical region provides the structural framework that constrains the quantum region.

### 3.4. Decoherence and Environmental Protection

In biological environments, quantum coherence is rapidly lost through interactions with the surrounding medium—a process called decoherence [26]. This explains why larger molecules generally behave classically. However, there is ongoing research into whether certain biological systems may have evolved to protect quantum coherence. Studies of photosynthetic light-harvesting complexes such as the Fenna-Matthews-Olson (FMO) complex in green sulfur bacteria have suggested quantum coherence might persist for hundreds of femtoseconds at physiological temperatures—longer than would be expected in comparable non-biological systems [27]. While the extent and functional significance of these quantum coherence effects remain subjects of scientific debate, they have inspired research into light-activated drugs such as photodynamic therapy agents [28].

### 3.5. Measurement Problems in Drug–Target Interactions

Heisenberg’s uncertainty principle has practical implications for measuring and predicting drug–target interactions. Nuclear magnetic resonance (NMR) spectroscopy, essential for drug discovery, is fundamentally limited by quantum uncertainty. When using NMR to determine protein–ligand binding conformations, the energy-time uncertainty relation (ΔE Δt ≥ ħ/2) creates a fundamental resolution limit [29]. This uncertainty is not just a technical limitation but a manifestation of quantum principles affecting our ability to characterize drug binding precisely.

Fragment-based drug discovery explicitly accounts for this uncertainty by using multiple weak-binding fragments rather than attempting to design a single perfectly fitting molecule. The drug vemurafenib (for melanoma treatment) was developed using this approach, acknowledging that quantum uncertainty makes perfect binding prediction impossible [30].

### 3.6. Boltzmann Distribution and Quantum States

The Boltzmann distribution determines the thermal population of quantum states in biological systems, creating a bridge between quantum mechanics and thermodynamics. The antihistamine fexofenadine (Allegra) binding to the histamine H1 receptor occurs through a conformational selection mechanism governed by the Boltzmann distribution [31]. Different receptor conformations exist in a thermal equilibrium described by:

A crucial concept often overlooked in discussions of quantum applications to biology is the Boltzmann distribution, which gives the probability of a particular state as a function of energy and temperature:

P(Ei)=∑je−Ej/kTe−Ei/kT

where P(Ei) is the probability of a state with energy Ei; k is the Boltzmann constant; T is the temperature

While quantum mechanical electron distributions determine each conformation’s energy (Ei), the probability of finding the receptor in a particular conformation (P(Ei)) follows this classical statistical distribution. Drug design must account for the quantum mechanical aspects of each conformation and the statistical thermodynamic distribution between conformations [32].

These examples illustrate how quantum effects, while not occurring at the molecular level as whole-molecule phenomena, nevertheless provide the fundamental foundation upon which molecular interactions relevant to drug discovery are built. The challenge—and opportunity—in modern pharmaceutical science lies in understanding how these quantum foundations propagate upward to influence observable molecular behavior and how drug design can exploit these effects for therapeutic advantage [33].

## 4. Historical Development of Quantum Applications in Drug Discovery

### 4.1. Pioneers in Quantum Biochemistry

While Schrödinger and Heisenberg laid the theoretical foundations in the 1920s, the practical application of quantum mechanics to biological problems began significantly later. Among the first pioneers were Bernard and Alberte Pullman, whose work in the 1950s at the Institut de Biologie Physico-Chimique in Paris established the field of quantum biochemistry [34]. Their 1963 book “Quantum Biochemistry” [6] and earlier works “Les Théories Electroniques de la Chimie Organique” (1952) and “Cancérisation par les substances Chimiques et Structure Moléculaire” (1955) [34] represented groundbreaking applications of quantum chemical principles to biological problems. Particularly notable was their work on predicting carcinogenic properties of aromatic hydrocarbons based on their electronic structure, one of the first successful applications of quantum theory to a medical problem [5].

Another key figure was Linus Pauling, whose 1935 book “Introduction to Quantum Mechanics—With Applications to Chemistry” (with E. Bright Wilson) [35] helped establish quantum chemistry as a discipline. Pauling’s work on the nature of the chemical bond, which earned him the 1954 Nobel Prize in Chemistry, applied quantum mechanical principles to explain molecular structure and provided a foundation for later work in computational drug design [16]. Per-Olov Löwdin and Charles Alfred Coulson also contributed significantly to developing quantum approaches in biochemistry during this period, establishing methodologies that would later become essential in drug discovery [36].

### 4.2. Computational Advances and the Rise of In Silico Drug Design

Despite these early theoretical foundations, practical applications in drug discovery were limited by computational constraints until the latter part of the 20th century. The 1990s marked a turning point with the development of more accessible and powerful computational tools, enabling the routine application of quantum mechanical calculations in pharmaceutical research [37]. The development of density functional theory (DFT) methods proved particularly significant. The 1998 Nobel Prize in Chemistry awarded to Walter Kohn “for his development of the density-functional theory” and John Pople “for his development of computational methods in quantum chemistry” recognized how these approaches had revolutionized computational chemistry and made quantum calculations feasible for drug-sized molecules [38].

Further advances came with the development of hybrid quantum mechanics/molecular mechanics (QM/MM) methods, recognized by the 2013 Nobel Prize in Chemistry awarded to Martin Karplus, Michael Levitt, and Arieh Warshel “for the development of multiscale models for complex chemical systems” [39]. These approaches allowed researchers to apply quantum mechanical calculations to the most critical parts of a biological system (such as an enzyme active site) while treating the surrounding environment with more computationally efficient classical methods [24]. Today, quantum mechanical calculations are routinely incorporated into drug discovery pipelines, from virtual screening of compound libraries to optimization of lead compounds and prediction of ADMET (absorption, distribution, metabolism, excretion, and toxicity) properties [40].

## 5. Quantum Mechanics in Modern Drug Discovery

### 5.1. Understanding Drug–Target Interactions

Quantum mechanical methods provide unprecedented insight into the interactions between drugs and their biological targets. Researchers can identify key interactions that drive binding affinity and selectivity by calculating the electronic structure of both the drug and the target [41]. These methods can reveal how changes in the electronic structure of a drug molecule might affect its binding to a protein target, guiding medicinal chemists in designing more potent and selective compounds [42]. Quantum approaches can also identify potential hydrogen bonding interactions, π-π stacking, and other non-covalent interactions critical for drug binding but difficult to predict with classical mechanical approaches [43].

Recent advances in computational power have enabled more accurate quantum mechanical calculations of larger systems, allowing researchers to model entire protein binding pockets rather than just simplified fragments. This has led to improved predictions of binding affinities and has helped identify new binding modes that might not have been apparent from classical molecular modeling [44]. These advances have been particularly valuable in structure-based drug design, where understanding the electronic details of drug–target interactions is essential for optimizing lead compounds.

### 5.2. Quantum Effects in Enzyme Catalysis

Enzymes are crucial biological catalysts and frequent targets for drug design. Quantum mechanical effects, particularly quantum tunneling, play a significant role in many enzymatic reactions involving hydrogen transfer, including proton, hydrogen atom, and hydride transfers [19]. Quantum tunneling allows particles to pass through energy barriers rather than over them, enabling reactions to proceed faster than would be predicted by classical transition state theory. Numerous enzymes have observed this phenomenon, including alcohol dehydrogenase, soybean lipoxygenase, and methylamine dehydrogenase [45,46].

Understanding these quantum effects is essential for designing effective enzyme inhibitors as drugs. Researchers can develop inhibitors that disrupt these processes with high specificity by targeting the specific electronic and structural features that enable quantum tunneling [47]. This approach has been particularly valuable in the design of drugs targeting enzymes involved in cellular respiration, DNA replication, and other critical biological processes [48]. Recent studies have shown that considering quantum tunneling effects can lead to the design of more effective inhibitors with improved selectivity and reduced side effects.

### 5.3. Density Functional Theory in Drug Design

Density functional theory (DFT) has emerged as one of the most powerful and widely used quantum mechanical methods in drug discovery. Rather than attempting to solve the complete Schrödinger equation for a many-electron system, DFT reformulates the problem regarding electron density, making calculations for drug-sized molecules computationally feasible [49]. DFT calculations provide valuable insights into molecular properties relevant to drug action, including electronic structure and charge distribution, binding energies and interaction strengths, conformational preferences and flexibility, reactivity and stability, and spectroscopic properties for experimental validation.

These calculations guide medicinal chemists in optimizing lead compounds, helping to improve properties such as potency, selectivity, and metabolic stability [50]. DFT has been particularly valuable in understanding metal-containing drug targets, such as metalloproteases and cytochrome P450 enzymes, where classical molecular mechanics often fail to accurately model the electronic interactions around metal centers [9]. Recent advances in DFT methods, including the development of dispersion-corrected functionals, have further improved the accuracy of these calculations for drug design applications [51]. These methods better account for van der Waals interactions, which are critical for drug binding but were historically challenging to model with DFT [11].

## 6. Quantum Tunneling in Drug Action and Development

### 6.1. Fundamentals of Quantum Tunneling

Quantum tunneling refers to the phenomenon where a particle passes through an energy barrier that, according to classical physics, cannot be overcome. This effect arises directly from the wave-like nature of quantum particles and the probabilistic interpretation of the wave function in the Schrödinger equation [52]. In classical physics, a particle with energy lower than a barrier’s potential energy cannot pass through the barrier. However, in quantum mechanics, the wave function of a particle extends beyond its classical boundaries, giving a non-zero probability of finding the particle on the other side of the barrier.

**Figure 2:**
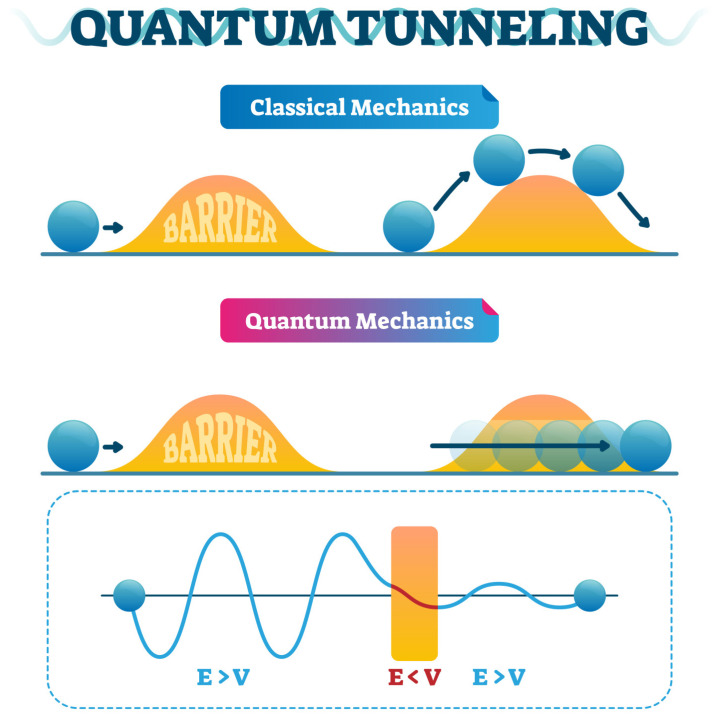
Visualization of quantum tunneling through a barrier (https://www.shutterstock.com/image-vector/quantum-tunneling-vector-illustration-infographic-classical-1180795456 (accessed on 25 April 2025).

Quantum tunneling is one of the properties of quantum mechanics that refers to the non-zero probability that a particle can be measured to be in a state forbidden in classical mechanics. Quantum tunneling occurs because a nontrivial solution exists for the Schrödinger equation in a classically forbidden region, corresponding to the exponential decay of the magnitude of the wave function. It plays a vital role across various fields, from the fundamental workings of stars and biological processes to cutting-edge technology in electronics and computing. However, its application in drug discovery is a relatively newer approach that still has many associated misconceptions due to the ethereal nature of the theory. For example, quantum tunneling arises from particles’ wave-like nature, determining the probability of finding the particle at a given position. In classical physics, a particle encountering an energy barrier higher than its kinetic energy would be unable to cross the barrier. For example, imagine a ball trying to roll over a hill; it will simply roll back if it does not have enough energy. When particles are not confined to a single point but exist as probability waves, they do not stop when they approach a barrier; instead, they decay exponentially inside it, giving a small but non-zero probability of the particles being found on the other side of the barrier with the same energy, making it look like a pass-through although appearing to violate classical physics depicted in Figure 2, where the wave function’s amplitude diminishes within the barrier but remains finite, allowing the particle to emerge on the other side.

It is worth pointing out that the particle’s probability of being in a space region is proportional to the square of the amplitude of its wave function in that region and that the particle’s energy is inversely proportional to its wavelength. It would also be worth indicating that the amplitude is lower to the right of the barrier because the barrier reflects part of the particle beam.

**Figure 3:**
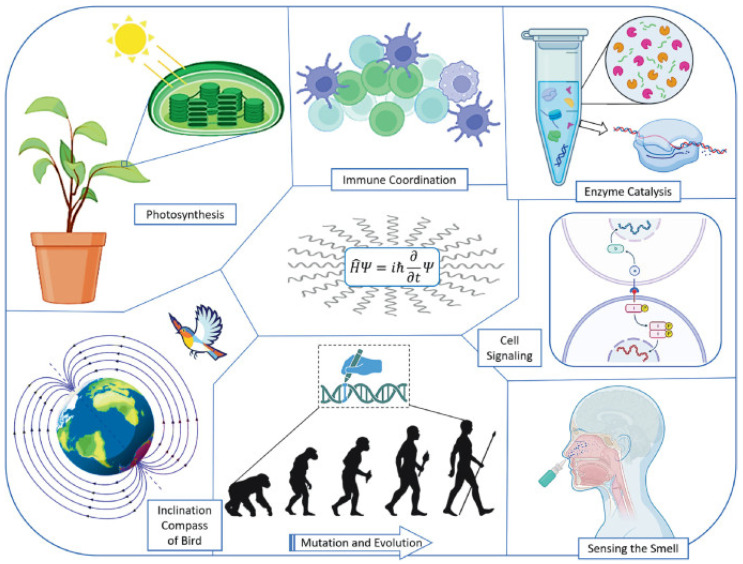
Quantum tunneling examples. (source Shutterstock).

One of the most remarkable examples of quantum tunneling occurs in photosynthesis, particularly within chlorophyll molecules found in plants. In photosynthesis, chlorophyll absorbs sunlight and uses its energy to drive the conversion of carbon dioxide and water into glucose and oxygen. At the heart of this process is the quantum coherence and tunneling of electrons within the chlorophyll, allowing for highly efficient energy transfer. When light strikes chlorophyll, it excites electrons to a higher energy state. These excited electrons need to move through a complex protein structure called the photosystem to reach the reaction center, where their energy is used to drive chemical reactions. Instead of moving through a direct path, electrons exploit quantum tunneling and coherence, allowing them to bypass energy barriers and find the most efficient route almost instantaneously. This quantum effect makes photosynthesis incredibly efficient, ensuring minimal energy is lost during the transfer process, which is far more effective than any available human-made solar technology [10]. Figure 3 illustrates how quantum tunneling works in chlorophyll molecules during photosynthesis and in a tunnel diode.

This figure illustrates various biological phenomena where quantum tunneling is believed to play a significant role. The time-dependent Schrödinger equation is at the center, symbolizing the quantum mechanical foundation underlying these processes. In photosynthesis, quantum coherence and tunneling enable the highly efficient transfer of excitonic energy through pigment-protein complexes [15]. Immune coordination may involve tunneling-mediated proton or electron transfer in redox signaling pathways essential for immune response. Enzyme catalysis exploits the quantum tunneling of protons or electrons across potential energy barriers, enhancing reaction rates beyond classical predictions. Cell signaling processes such as neurotransmission may involve tunneling during receptor–ligand interactions or ion transport across membranes. The olfactory system (sensing smell) has been proposed to use inelastic electron tunneling, where odorant molecules are identified via their vibrational spectra. Mutation and evolution can be influenced by proton tunneling in DNA base pairs, leading to spontaneous mutations that drive evolutionary change. Bird navigation is explained by the inclination compass hypothesis, where entangled electron spins in cryptochrome proteins respond to Earth’s magnetic field—a quantum biological compass. These diverse phenomena collectively highlight the pervasive role of quantum tunneling in life processes, often enabling biologically essential reactions and behaviors that classical physics alone cannot fully explain.

The molecules responsible for the light-to-energy conversion are chromophores, otherwise known as chlorophyll, and they rely on dipole coupling. This is when two molecules do not share their electrons evenly but instead have an unbalanced charge difference. This difference allows electrons to flow to the positively charged side, generating electricity during the process. These diploes exist in the chlorophyll, and with the light being converted into energy, the electrons are free to flow along the membranes and allow the necessary chemical reactions that plants need to break down CO_2_.

The quantum part is derived from the dipoles experiencing entanglement, or particles can change each other’s state without physical contact. A classic example would be having two cards of different colors flipped upside down. If I draw one color, I know the color of the other without doing anything to it. With chlorophyll, factors like surrounding molecules and orientation can influence this entanglement with other particles in the system.

The tunneling is particularly relevant in drug design when targeting biochemical reactions that rely on proton or electron transfers [21]. For example, enzymes facilitating these transfers often utilize tunneling as a catalytic mechanism [10]. By incorporating the Schrödinger equation, researchers can design inhibitors that specifically enhance or disrupt these tunneling pathways, potentially leading to more effective drugs for modulating the activity of key enzymes involved in diseases such as cancer or metabolic disorders [9]. In antiviral and antibacterial drug design, targeting proton pumps or polymerases where tunneling plays a role can lead to drugs specifically tailored to exploit or block these unique quantum effects, potentially overcoming resistance mechanisms that rely on traditional binding interactions [9].

Tunneling is also relevant in drug-binding interactions where electron transfer stabilizes the drug–target complex, such as in specific receptor–ligand systems or redox-active binding sites [50]. For instance, drugs designed to enhance hydrogen bonding networks through proton tunneling can achieve more substantial and specific interactions, particularly useful in developing therapies for neurological and cardiovascular diseases [53].

The tunneling probability decreases exponentially with increasing barrier width, particle mass, and the difference between barrier height and particle energy. This explains why tunneling is most significant for light particles like electrons and hydrogen atoms and less relevant for heavier atoms and molecular groups [27]. This fundamental quantum mechanical phenomenon has profound implications for understanding chemical reactions in biological systems, particularly those involving the transfer of light particles such as protons and electrons.

### 6.2. Tunneling in Enzymatic Reactions

In biological systems, quantum tunneling is particularly important in enzymatic reactions involving hydrogen transfer, including proton, hydrogen atom, and hydride transfers [19]. These reactions are central to many metabolic processes and are frequently targeted in drug design. Several enzymes show clear evidence of hydrogen tunneling, including alcohol dehydrogenase, which catalyzes the interconversion of alcohols and aldehydes or ketones [45]; soybean lipoxygenase, which catalyzes the deoxygenation of polyunsaturated fatty acids [46]; methylamine dehydrogenase, which oxidizes methylamine to formaldehyde [54]; and cytochrome P450 enzymes, which are crucial for drug metabolism [55].

Tunneling in these enzymes is typically identified through measurement of kinetic isotope effects (KIEs). Replacing hydrogen with deuterium or tritium should result in predictable changes in reaction rates based on classical transition state theory. However, many enzymes show KIEs larger than classical predictions and exhibit anomalous temperature dependencies, providing strong evidence for tunneling [56]. Modern enzymatic hydrogen tunneling theories emphasize the role of protein dynamics in facilitating the process. Specific motions within the enzyme can transiently reduce the width of the barrier, significantly increasing the tunneling probability [48]. These motions are often precisely optimized through evolution to maximize catalytic efficiency.

### 6.3. Implications for Drug Design

Understanding quantum tunneling in enzymatic reactions has significant implications for drug design, particularly for inhibitors of enzymes involving hydrogen transfer [57]. Traditional approaches to enzyme inhibitor design focus on mimicking transition states or binding to the active site. However, a more sophisticated approach could disrupt the specific protein motions that facilitate tunneling, potentially leading to more selective inhibitors with fewer side effects [58].

For example, in designing inhibitors for cytochrome P450 enzymes, which are crucial for drug metabolism, consideration of tunneling effects could lead to compounds that specifically disrupt the hydrogen transfer step without affecting other functions of these versatile enzymes [59]. Similarly, understanding tunneling in proton transfer reactions could inform the design of proton pump inhibitors, which are widely used to treat conditions such as gastroesophageal reflux disease and peptic ulcers [60].

In addition to inhibitor design, quantum tunneling might be exploited to enhance drug activity. Compounds designed to promote specific tunneling pathways in target enzymes could act as molecular switches, activating desired functions while minimizing side effects [61]. This approach represents a novel paradigm in drug design that moves beyond the traditional lock-and-key model to consider biological systems’ dynamic, quantum mechanical nature.

## 7. Applications Across Therapeutic Areas

### 7.1. Neurological Drugs

The central nervous system presents unique challenges and opportunities for quantum-informed drug design. Neurotransmitter–receptor interactions, ion channel function, and synaptic transmission all involve processes that can be influenced by quantum effects [62]. Quantum tunneling may play a role in the rapid movement of ions through channel proteins, affecting neural signaling. This understanding could inform the design of more effective drugs for conditions such as epilepsy, neuropathic pain, and neurodegenerative diseases [63].

For example, in designing drugs targeting voltage-gated sodium channels for pain management, considering quantum effects in ion transport could lead to compounds with improved selectivity and reduced side effects [64]. Similarly, understanding the quantum aspects of neurotransmitter-receptor binding could inform the development of more effective treatments for psychiatric disorders. Recent studies suggest that quantum effects might influence the binding of neurotransmitters such as serotonin and dopamine to their receptors, potentially explaining some of the complex pharmacology of these systems [65].

### 7.2. Oncology Drugs

Cancer treatment is another area where quantum mechanical approaches have made significant contributions. Quantum calculations have been essential in understanding the interactions between anticancer drugs and their targets, particularly DNA and key enzymes involved in cell division [53]. For example, quantum mechanical studies of platinum-based drugs such as cisplatin have revealed how these compounds bind to DNA and disrupt its function, leading to cancer cell death. These insights have guided the development of next-generation platinum drugs with improved efficacy and reduced toxicity [66].

Quantum tunneling may also play a role in specific anticancer mechanisms. Some DNA repair enzymes utilize electron transfer reactions that likely involve tunneling, and disrupting these processes could sensitize cancer cells to DNA-damaging therapies [67]. More recently, quantum mechanical calculations have been crucial in designing targeted cancer therapies, such as tyrosine kinase inhibitors. Researchers can optimize binding affinity and selectivity by accurately modeling the electronic structure of the drug and the target, leading to more effective and less toxic treatments [68].

### 7.3. Cardiovascular Drugs

Cardiovascular diseases remain a leading cause of morbidity and mortality worldwide, and quantum mechanical approaches have contributed significantly to drug development in this area [69]. Quantum calculations have been particularly valuable in understanding the interactions between drugs and ion channels that regulate cardiac rhythm. By accurately modeling the electronic structures involved in these interactions, researchers have developed more selective channel blockers with fewer side effects [70].

Similarly, quantum mechanical studies of angiotensin-converting enzyme (ACE) inhibitors have provided insights into their mechanism of action and guided the development of improved compounds for treating hypertension and heart failure [71]. Recent work has also explored the quantum aspects of nitric oxide signaling, which is crucial for vascular function. Understanding the electronic structure of nitric oxide and its interactions with target proteins has informed the development of drugs that modulate this pathway for treating conditions such as pulmonary hypertension [72].

### 7.4. Antimicrobial Drugs

The growing challenge of antimicrobial resistance has heightened the need for new approaches to antibiotic development, and quantum mechanical methods are playing an increasingly important role in this field [73]. Quantum calculations have been essential in understanding the mechanisms of action of existing antibiotics and identifying potential new targets. For example, detailed modeling of β-lactam antibiotics and their target enzymes has revealed how resistance mutations alter the electronic structure of binding sites, informing the design of new compounds that can overcome these resistance mechanisms [74].

Quantum tunneling may also be relevant to specific antimicrobial mechanisms. Some antibiotics that target cell wall synthesis involve hydrogen transfer reactions that tunneling effects could influence, and understanding these processes could lead to more effective compounds [75]. More recently, quantum mechanical approaches have been applied to design novel antimicrobial peptides, representing a promising alternative to traditional antibiotics. By accurately modeling the electronic interactions between these peptides and bacterial membranes, researchers are developing compounds with improved selectivity and reduced potential for resistance development [76].

## 8. Computational Methods and Challenges

### 8.1. Hybrid QM/MM Approaches

A key development in applying quantum mechanics to drug discovery has been the hybrid quantum mechanics/molecular mechanics (QM/MM) approach [77]. This method, recognized with the 2013 Nobel Prize in Chemistry, allows researchers to model large biological systems by treating the most critical parts (such as an enzyme active site or drug binding pocket) with quantum mechanics while using more computationally efficient classical mechanics for the surrounding environment [39]. QM/MM approaches have been particularly valuable for studying enzymatic reactions and drug–target interactions, where quantum effects are localized but influenced by the broader protein environment [24]. By combining the accuracy of quantum calculations with the efficiency of molecular mechanics, these methods provide a practical way to apply quantum mechanical principles to drug discovery.

Recent advances in QM/MM methodologies include the development of more sophisticated boundary treatments between QM and MM regions, the implementation of polarizable force fields that better capture electronic responses to changes in the environment, integration with enhanced sampling techniques to explore conformational space more efficiently, and improvements in energy correction schemes to address systematic errors. These advances have expanded the scope and accuracy of QM/MM calculations in drug discovery applications [78].

### 8.2. Time-Dependent Density Functional Theory

While standard DFT calculations are valuable for studying ground state properties, many drug interactions involve excited states or time-dependent processes. Time-dependent density functional theory (TD-DFT) extends DFT to these dynamic situations, allowing researchers to model spectroscopic properties, photochemical reactions, and electron transfer processes relevant to drug action [79]. TD-DFT has been particularly valuable for studying fluorescent properties of drug molecules for imaging applications, photodynamic therapy agents that generate reactive oxygen species upon light activation, electron transfer processes in redox-active drugs, and excited state properties of biological chromophores that drugs might target.

Despite its utility, TD-DFT faces challenges in accurately modeling certain excited states, particularly those with charge-transfer character. Ongoing development of improved functionals and computational approaches continues to address these limitations [80]. These developments are essential for expanding the application of quantum mechanical methods to drugs that interact with biological systems through excited state processes or electron transfer mechanisms.

### 8.3. Machine Learning and Quantum Mechanics

Integrating machine learning with quantum mechanical calculations represents one of the most promising recent developments in computational drug discovery [51]. Machine learning algorithms can be trained on quantum mechanical data to predict properties of new compounds with much greater computational efficiency, potentially accelerating the drug discovery process. Several approaches have been developed to combine machine learning with quantum mechanics: neural networks trained to predict quantum mechanical energies and properties based on molecular structure, kernel methods that learn mappings between molecular descriptors and quantum properties, deep learning models that generate novel molecular structures with desired quantum properties, and transfer learning approaches that apply knowledge from small molecules to larger drug-like compounds.

These machine-learning models can achieve near-quantum mechanical accuracy with computational costs orders of magnitude lower than direct calculations, making quantum-level predictions feasible for high-throughput virtual screening and lead optimization [81]. For example, recent work has demonstrated machine learning to predict coupled-cluster quantum chemical energies from DFT calculations, achieving quantum chemical accuracy (errors below 1 kcal·mol⁻¹) with computational costs similar to standard DFT [82].

### 8.4. Current Limitations and Future Directions

Despite significant advances, several challenges remain in applying quantum mechanical principles to drug discovery. These include the computational cost of fully quantum mechanical treatments of large biological systems, the accuracy of approximations used in practical quantum chemical methods, the treatment of complex dynamics over multiple time scales, the integration of quantum mechanical insights with experimental data, and the quantification of uncertainty in quantum mechanical predictions [40].

Future directions to address these challenges include the development of more efficient algorithms and computational approaches, potentially leveraging emerging quantum computing technologies, improved integration of quantum mechanical calculations with multiscale modeling approaches, enhanced methods for sampling conformational space and modeling dynamics, better incorporation of quantum mechanical insights into machine learning models, and development of more accurate and transferable force fields informed by quantum mechanical calculations [83]. These advances promise to enhance further the impact of quantum mechanical principles on drug discovery in the coming years.

## 9. Additional Quantum Concepts for Drug Discovery

While the preceding sections have covered several fundamental quantum mechanical principles relevant to drug discovery, recent research has identified additional quantum concepts with significant promise for pharmaceutical science. These concepts further illustrate how quantum mechanics continues to provide new opportunities for drug design and development beyond the traditional applications of the Schrödinger equation and the uncertainty principle.

### 9.1. Quantum Delocalization and Aromaticity

Quantum delocalization of electrons underlies the concept of aromaticity, which is critical in drug design. Many pharmaceutical compounds contain aromatic rings whose stability and reactivity are governed by quantum mechanical principles of electron delocalization. This principle dates to Hückel’s work in the 1930s but remains central to modern drug design [84]. Aromaticity arises from the quantum mechanical phenomenon of electron delocalization, where electrons are distributed across multiple atoms rather than confined to specific bonds. This delocalization leads to increased stability and distinctive chemical properties that are exploited in drug design. Quantifying aromaticity through quantum mechanical calculations provides essential insights for medicinal chemists designing compounds with specific electronic properties.

For example, many drugs targeting protein–protein interactions contain aromatic rings that engage in π-π stacking interactions with aromatic amino acid residues in the target protein. The strength and geometry of these interactions can only be accurately predicted through quantum mechanical calculations that account for electron delocalization effects [85]. Similarly, the design of heterocyclic compounds, which form the backbone of many drug classes, including benzodiazepines, quinolones, and azoles, relies on understanding how quantum delocalization affects their electronic properties and reactivity.

Recent advances in computational methods have enabled more accurate predictions of aromaticity and its effects on drug properties, developing novel therapeutic agents with optimized electronic structures [86].

### 9.2. Quantum Phase Transitions in Allosteric Modulation

Recent research (2021–2024) suggests that allosteric modulation in proteins may involve quantum phase transitions, where small changes in ligand binding can propagate through quantum mechanical effects to cause large-scale conformational changes. This concept is particularly relevant for designing allosteric modulators as drugs, representing a growing class of therapeutics [87]. Quantum phase transitions occur when a system undergoes an abrupt change in its quantum state as a parameter varies continuously. In the context of protein allostery, the binding of a ligand at one site may induce changes in the quantum state of the protein that propagates to distant sites, affecting its function. This quantum perspective on allostery goes beyond classical mechanical models and may explain allosteric regulation’s remarkable sensitivity and efficiency in biological systems.

Studies of G-protein-coupled receptors (GPCRs), essential targets for approximately 30% of approved drugs, have revealed evidence of quantum phase transition-like behavior in their activation mechanisms [88]. By understanding these quantum effects, researchers may design more effective allosteric modulators that can precisely control receptor activation states, potentially leading to drugs with fewer side effects and greater therapeutic specificity. Computational approaches to modeling quantum phase transitions in proteins are still developing, but they represent a promising frontier for structure-based drug design targeting allosteric sites [89].

### 9.3. Non-Adiabatic Dynamics in Drug–Target Interactions

Non-adiabatic dynamics, where electronic and nuclear motions couple in ways that violate the Born–Oppenheimer approximation, have emerged as essential factors in understanding drug–target interactions. Recent studies (2022–2023) show that non-adiabatic effects influence drug binding kinetics and can be manipulated to design drugs with specific on/off rates [90]. The Born-Oppenheimer approximation, which separates electronic and nuclear motions based on their vastly different timescales, is a cornerstone of most quantum chemical calculations. However, in certain situations, particularly when electronic states come close in energy, this approximation breaks down, and non-adiabatic effects become significant. These effects can influence the rates and mechanisms of chemical processes relevant to drug action.

For example, proton and electron transfer reactions involved in drug metabolism often exhibit non-adiabatic behavior, where the coupling between electronic and nuclear motions significantly affects reaction rates [91]. Understanding these non-adiabatic effects can lead to more accurate drug metabolism and toxicity predictions, which are crucial aspects of drug development. Similarly, recent research has shown that non-adiabatic dynamics can influence drug binding kinetics, particularly the residence time of a drug at its target, which is increasingly recognized as a critical parameter for drug efficacy [80]. By designing drugs that exploit specific non-adiabatic pathways, medicinal chemists may be able to optimize binding kinetics for improved therapeutic outcomes.

### 9.4. Quantum Confinement Effects in Nanomedicine

As drug delivery increasingly utilizes nanoparticles, quantum confinement effects become relevant, where particle properties change due to spatial restriction. These quantum mechanical phenomena govern the properties of quantum dots and nanoparticles used in targeted drug delivery, connecting fundamental physics to cutting-edge pharmaceutical technologies [92]. Quantum confinement occurs when the size of a particle approaches the wavelength of its electronic wave function, leading to discrete energy levels and size-dependent properties. This phenomenon is particularly prominent in semiconductor nanoparticles (quantum dots), where the band gap energy increases as particle size decreases, leading to size-tunable optical and electronic properties.

In nanomedicine, these quantum confinement effects are exploited for imaging, sensing, and drug delivery applications. Quantum dots can be fluorescent labels for tracking drug delivery with exceptional brightness and photostability [93]. More sophisticated applications include using quantum confinement effects to create stimuli-responsive nanocarriers that release drugs in response to specific triggers or to design nanoparticles that can convert light or other forms of energy into therapeutic effects through quantum-confined structures [94]. The design of these nanomedicine platforms requires a sophisticated understanding of quantum mechanics to predict and optimize the properties of confined systems. Computational methods that can accurately model quantum confinement effects in complex biological environments are becoming essential tools in nanomedicine development.

### 9.5. Vibrational Strong Coupling in Drug Activity

An emerging area (2020–2024) explores how strong vibrational coupling between molecular vibrations and electromagnetic cavity modes can alter chemical reactivity. This quantum effect has potential applications in controlling drug metabolism and activation, representing a novel avenue for pharmaceutical research [95]. Vibrational strong coupling occurs when a molecular vibration interacts strongly with the confined electromagnetic field of an optical cavity, leading to the formation of hybrid light-matter states called polaritons. These polaritons have properties distinct from the original molecular vibration and the cavity mode, including altered energy levels and symmetry properties that can influence chemical reactivity.

Recent experimental work has demonstrated that vibrational strong coupling can modify chemical reaction rates and selectivity without any direct excitation of the system, suggesting a novel approach to controlling chemical processes [96]. In the context of drug discovery, this could lead to innovative methods for modulating drug activity, stability, or metabolism. For example, strong vibrational coupling could potentially be used to protect labile drugs from degradation, control the release of prodrugs, or develop novel activation mechanisms for targeted therapy [97]. While still in its early stages, this research area represents a fascinating convergence of quantum optics and pharmaceutical science with potential long-term implications for drug development.

Integrating these quantum concepts into drug discovery workflows promises to enrich our understanding of molecular interactions and open new avenues for therapeutic intervention. As computational methods advance and experimental techniques become more sophisticated, these quantum principles will likely play an increasingly important role in developing next-generation pharmaceuticals.

## 10. Future Perspectives: Quantum Biology and Medicine

### 10.1. Quantum Coherence in Biological Systems

Beyond tunneling, other quantum phenomena, such as quantum coherence and entanglement, may play roles in biological systems [13]. Quantum coherence refers to the ability of quantum systems to exist simultaneously in multiple states. It has been hypothesized to play a role in photosynthesis, potentially contributing to the remarkably efficient energy transfer observed in light-harvesting complexes [27]. However, it is important to note that quantum coherence’s extent and functional significance in biological systems remain subjects of active scientific debate and investigation.

Recent research has explored whether specific biological systems might have evolved to protect and exploit quantum coherence, even in warm and noisy cellular environments where quantum effects would typically be expected to decohere rapidly [48]. These findings have prompted speculation about whether similar quantum coherence effects might be relevant to drug action or could be exploited in drug design. For example, understanding how biological systems maintain quantum coherence could inspire the design of drugs that modulate coherence-dependent processes or utilize coherence effects to enhance their efficacy or selectivity [33]. While this area remains highly theoretical, it represents an intriguing frontier for future drug discovery research.

### 10.2. Quantum Computing in Drug Discovery

The emergence of quantum computing represents a potentially transformative development for drug discovery. Quantum computers, which exploit quantum mechanical phenomena such as superposition and entanglement to perform calculations, have the potential to solve specific problems exponentially faster than classical computers [82]. Several applications of quantum computing in drug discovery are being explored:

Quantum simulations directly simulate quantum systems on quantum computers, potentially enabling exact solutions to the Schrödinger equation for drug-sized molecules [81]. Quantum machine learning uses quantum algorithms to enhance machine learning approaches for drug discovery, potentially improving predictive models for drug properties and interactions [82]. Optimization problems apply quantum optimization algorithms to challenges such as protein folding, molecular docking, and lead optimization [81].

While current quantum computers remain limited in their capabilities, rapid progress suggests that quantum computing may become an essential tool for drug discovery in the coming decades [82]. As quantum hardware advances, the potential applications of quantum computing in pharmaceutical research are likely to expand, potentially revolutionizing our approach to designing and developing new therapeutics.

### 10.3. Designing Drugs to Exploit Quantum Effects

Perhaps the most speculative but potentially transformative application of quantum principles in drug discovery involves designing drugs that specifically exploit quantum effects in their mechanism of action [13]. Some possibilities include tunneling-enhanced drugs, which are compounds designed to facilitate or inhibit specific tunneling pathways in biological targets, potentially offering unprecedented selectivity [57]; coherence-modulating drugs, which are molecules that influence quantum coherence in biological systems, potentially affecting processes such as photosynthesis or specific aspects of neural function [48]; and quantum switches, which are compounds that can exist in quantum superpositions of active and inactive states, potentially enabling novel modes of action or reduced side effects [33].

While these concepts remain largely theoretical, they represent intriguing possibilities for the future of drug discovery as our understanding of quantum biology advances [98]. As our ability to manipulate and control quantum systems continues to improve, we may eventually be able to design drugs that operate according to principles beyond classical molecular recognition, potentially leading to entirely new classes of therapeutics with unprecedented properties and capabilities.

## 11. The Quantum Future of Medicine

As we celebrate the centenary of the Schrödinger equation and Heisenberg’s uncertainty principle, reflecting on how these abstract mathematical formulations have evolved from pure theoretical physics to practical tools driving innovation in pharmaceutical science is remarkable. From the pioneering work of the Pullmans in the 1950s to today’s sophisticated quantum mechanical simulations and emerging quantum technologies, the journey has been one of continuous discovery and application.

The quantum perspective has fundamentally changed our understanding of drug action and design. Rather than viewing drugs as static molecules with fixed properties, we now recognize drug–target interactions’ dynamic, probabilistic nature, influenced by quantum effects such as tunneling and coherence. This deeper understanding has led to more rational approaches to drug design, enabling the development of more effective and selective therapeutics [99].

Our examination has revealed that quantum effects, despite occurring primarily at atomic and subatomic scales, profoundly influence the behavior of larger molecular systems through localized quantum events, electron distributions, and collective phenomena. The practical application of quantum principles through methods like density functional theory, hybrid QM/MM approaches, and quantum-inspired machine learning algorithms has revolutionized our ability to design drugs with unprecedented precision [82].

Integrating quantum mechanical principles with advances in computational methods, experimental techniques, and emerging technologies such as quantum computing promises to accelerate drug discovery further. As our understanding of quantum biology grows, we may discover entirely new paradigms for therapeutic intervention, potentially addressing currently untreatable diseases. The continuing merger of Schrödinger’s wave mechanics, Heisenberg’s uncertainty principle, and Boltzmann’s statistical thermodynamics create a robust framework for exploring and exploiting quantum effects in biological systems, opening new frontiers in pharmaceutical science [100].

The story of quantum mechanics in drug discovery is a testament to the power of fundamental science to drive practical innovation. What began as abstract equations describing the behavior of subatomic particles has become an essential framework for understanding and manipulating the molecular machinery of life. As we look to the next century of quantum science, we can anticipate even more profound connections between quantum physics and medicine, continuing the remarkable journey that began with Schrödinger and Heisenberg a hundred years ago [101].

## 12. Philosophical and Ethical Limits of the Role of Quantum Mechanics

The quantum future of medicine calls for a deeper philosophical reflection that connects the mathematics we have mastered with the mysterious phenomena they describe. As we approach the conclusion of our examination of quantum mechanics’ role in pharmaceutical science over the past century, we must acknowledge that Schrödinger’s seminal work “What is Life?” [3] presciently anticipated the very bridge between quantum physics and biological sciences that we have documented throughout this review. When Schrödinger posed the question of how quantum-level physical laws might govern macroscopic biological phenomena, he initiated a profound intellectual journey that continues today [98]. The remarkable aspect of quantum mechanics—that we possess precise mathematical descriptions of phenomena whose fundamental nature eludes complete understanding—creates a unique epistemic position from which to develop therapeutics. We can predict and manipulate quantum effects without fully comprehending the underlying reality, which empowers and humbles the scientific enterprise [15]. This knowledge paradox mirrors the philosophical tension accompanying quantum theory since its inception: our most successful physical theory provides extraordinary predictive power while challenging our intuitive understanding of reality [99].

The transition from quantum equations to pharmaceutical innovations should not be viewed as a linear progression of purely technological developments occurring in isolation. Rather, this evolution represents a complex, interactive process that involves multiple knowledge domains, cultural contexts, and stakeholders beyond the laboratory setting [102]. As “Innovation Studies” scholars have long recognized, scientific advancement occurs through recursive feedback between theory and application, between discipline-specific and transdisciplinary approaches, and between specialists and broader societal needs [103]. The quantum–pharmaceutical interface exemplifies this interactive model, with advances in computational methods enabling practical applications of previously theoretical quantum concepts, which in turn generate new questions requiring theoretical refinement [104]. This dialectical relationship has accelerated in recent decades as computational power and algorithmic sophistication have grown exponentially, allowing pharmaceutical researchers to implement increasingly accurate quantum mechanical calculations in drug discovery workflows [105].

The non-linearity of scientific progress in this domain stems from computational constraints and the inherent complexity of transitioning between scales—from quantum effects at the subatomic level to emergent biological phenomena at cellular and systemic levels [106]. Bridging these scales requires careful epistemological navigation between reductionist and holistic approaches, acknowledging that specific properties of biological systems cannot be directly derived from quantum mechanical first principles alone [107]. This multiscale reality necessitates integrative methodologies that accommodate both bottom-up quantum calculations and top-down biological observations, creating frameworks that can translate between these different levels of description [83]. The challenge of scale-bridging in quantum biology echoes Schrödinger’s original inquiry about how quantum indeterminacy might be reconciled with the apparent determinism of biological processes—a question that remains philosophically rich even as we develop practical tools to address it technically [108].

As quantum approaches in medicine advance, we must also confront profound ethical considerations that extend beyond standard bioethical frameworks. The capacity to manipulate matter at its most fundamental level raises questions about whether natural boundaries exist that should limit our interventions in biological systems [109]. Unlike classical pharmaceutical approaches that typically modify existing biochemical pathways, quantum-based therapeutics might potentially alter the very physical foundations upon which biological processes operate [110]. This represents a quantitative and qualitative shift in our technological relationship with nature, requiring careful ethical reflection that draws from scientific understanding and humanistic wisdom [111]. Philosophical discussions about the ethics of quantum medicine must address questions of ontological significance: Are we merely extending existing medical paradigms, or are we fundamentally redefining the relationship between physics and biology in ways that demand new ethical frameworks [112]?

The ethical dimensions extend further when considering quantum computing applications in drug discovery, which promise unprecedented processing power but also raise questions about accessibility, knowledge ownership, and technological dependency [82]. As quantum technologies become essential tools for pharmaceutical innovation, ensuring equitable access becomes an ethical imperative to prevent widening global healthcare disparities [113]. Similarly, the increasing complexity and computational demands of quantum-based drug design could concentrate innovation capabilities among a few technologically advanced institutions, potentially limiting diverse approaches to therapeutic discovery [114]. These socio-economic considerations remind us that scientific advancement occurs within broader societal contexts that influence its direction and beneficiaries [115].

Perhaps the most profound philosophical question raised by the quantum-pharmaceutical interface concerns the nature of life itself. Schrödinger’s visionary work suggested that understanding life might require acknowledging its connection to fundamental physical principles while recognizing its unique organizational properties [116]. As we develop therapeutics that operate at the quantum level, we implicitly engage with this ontological question about whether living systems represent merely complex arrangements of quantum particles or embody emergent properties that transcend their physical constituents [117]. This philosophical tension between reductionism and emergence permeates modern quantum biology and informs how we conceptualize disease and healing [118]. By acknowledging these more profound questions, we enrich the scientific enterprise and ensure that our technological capabilities remain guided by thoughtful reflection on their meaning and purpose [119].

As we look toward the next century of quantum applications in pharmaceutical science, we must embrace what philosopher of science Thomas Kuhn identified as the essential tension in scientific progress—balancing traditional knowledge with transformative innovation [120]. The quantum revolution in drug discovery represents not merely a set of new techniques but a fundamentally different way of understanding molecular interactions, one that continues to validate Schrödinger’s intuition that quantum physics would eventually illuminate the mysteries of biological processes [33]. By consciously engaging with both the technical and philosophical dimensions of this scientific journey, we honor the visionary thinkers who established quantum theory while ensuring that its biomedical applications serve human flourishing in ethically sound ways [121]. The quantum future of medicine thus beckons us toward not only more effective therapeutics but also a deeper understanding of life itself—an understanding that emerges from the remarkable convergence of physical theory, biological observation, and philosophical reflection that Schrödinger himself envisioned nearly eight decades ago [122].

## Data Availability

No new data were created or analyzed in this study.
